# Epidémiologie de l’association fibrillation atriale et insuffisance cardiaque

**DOI:** 10.11604/pamj.2017.26.116.11470

**Published:** 2017-03-02

**Authors:** Yassine Ragbaoui, Chafia Chehbouni, Ayoub El Hammiri, Rachida Habbal

**Affiliations:** 1 Service de Cardiologie, Centre Hospitalier Universitaire IBN ROCHD, Casablanca, Maroc

**Keywords:** Fibrillation atriale, insuffisance cardiaque, prévalence, Atrial fibrillation, Heart failure, prevalence

## Abstract

La fibrillation atriale et l’insuffisance cardiaque sont des maladies cardiovasculaires souvent associées, leur coexistence aggrave le pronostic et constitue un problème de santé public majeur. L’objectif de cette étude est d’évaluer la prévalence de la fibrillation atriale chez les patients en insuffisance cardiaque chronique et déterminer le profil clinique de ce groupe de patients. Nous avons conduit une étude rétrospective descriptive basée sur le registre d'insuffisance cardiaque du département de cardiologie du centre hospitalier universitaire de juin 2006 à Mars 2015.Nous avons inclus les patients âgés de plus de 18 ans ayant une insuffisance cardiaque chronique avec une fibrillation atriale. Durant cette période 3048 patients ayant une insuffisance cardiaque chronique avec fibrillation atriale(FA) ont été inclus. La prévalence de la fibrillation atriale était de 10,6%. La fréquence des facteurs de risque cardiovasculaires associée à (FA) était de: (54%) pour l'hypertension artérielle, (39%) pour le diabète, (8%) pour la dyslipidémie, (26%) pour le tabac, (30%) pour la sédentarité et (17%) pour la ménopause. La (FA) était présente chez (67,4%) des hommes versus (32,6%) des femmes. Les étiologies de l'insuffisance cardiaque chronique associées à la fibrillation atriale étaient: les valvulopathies (44,1%); les coronaropathies (32%); Hypertensives (11%), primitive (10%), toxiques (2%) et alcoolisme chronique (1%). La fibrillation atriale reste fréquente dans la population des patients insuffisants cardiaques au Maroc et la moyenne d’âge est inférieure à celle retrouvée dans la littérature.

## Abstract (en)

Atrial fibrillation and heart failure are diseases which often coexist aggravating prognosis and constituting a major public health problem. The aim of this study was to assess the prevalence of atrial fibrillation in patients with chronic heart failure and to determine the clinical profile of this group of patients. We conducted a retrospective descriptive study based on the registry of heart failure of the Department of Cardiology at the University Hospital Centre from June 2006 to March 2015. All patients over 18 years of age with chronic heart failure associated with atrial fibrillation were included in the study. During the study period 3048 patients with chronic heart failure associated with atrial fibrillation (AF) were enrolled. The prevalence of heart failure was 10.6%. The frequency of cardiovascular risk factors associated with (AF) was: (54%) hypertension, (39%) diabetes, (8%) dyslipidemia, (26%) tobacco, (30%) sedentary lifestyle and (17%) menopause. FA affected 67.4% of men and 32.6% of women. The causes of chronic heart failure associated with atrial fibrillation were: heart valve diseases (44;1%); coronary artery diseases (32%); hypertensive (11%), primary (10%), toxic (2%) and chronic alcoholism (1%). Atrial fibrillation frequently appears in patients with heart failure in Morocco and it occurs at an average age lower than that found in the literature.

## Introduction

La fibrillation atriale (FA) et l’insuffisance cardiaque (IC) sont des maladies cardiovasculaires souvent associées, leur coexistence aggrave le pronostic et constitue un problème de santé public majeur [1]. La prévalence de la FA dans la population générale est estimée à un 1%, elle augmente significativement chez les sujets âgées [2]. L’incidence de la FA va probablement doubler dans les 20 prochaines années [3] d’une part en raison de l’augmentation de la longévité et d’autre part en raison de la réduction de la mortalité cardiovasculaire. La prévalence de la fibrillation atriale et son épidémiologie spécialement chez des patients en insuffisance cardiaque chronique reste mal connue dans notre pays malgré la gravité de son pronostic et son cout excessive sur le système des soins.

## Méthodes

Nous avons conduit une étude rétrospective descriptive basée sur le registre d'insuffisance cardiaque du département de cardiologie du centre hospitalier universitaire IBN ROCHD à Casablanca de juin 2006 à Mars 2015. Nous avons inclus les patients des deux sexes âgés de plus de 18 ans admis pour une insuffisance cardiaque chronique affirmée sur les bases cliniques, biologiques, électrocardiographiques et confirmée par une échocardiographie Doppler ayant une fibrillation atriale associée. Nous avons exclu les patients atteints de cardiopathies congénitales. L'analyse statistique a été réalisée à l'aide d'un logiciel SPSS version 13.0 pour Windows.

## Résultats

**Figure 1:**
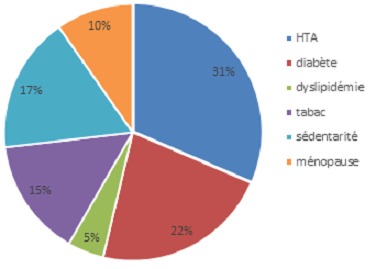
Facteurs de risque cardiovasculaire dans l’association insuffisance cardiaque et fibrillation atriale

**Figure 2:**
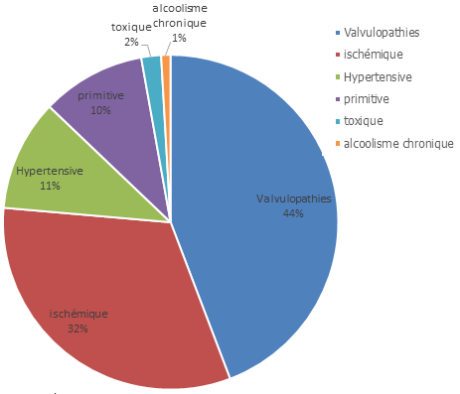
Étiologies de l’insuffisance cardiaque des patients en fibrillation atriale

3048 patients ont été inclus ayant une insuffisance cardiaque chronique (ICC) avec fibrillation atriale (FA). La prévalence de la fibrillation atriale était de 10,6%. La moyenne d’âge était de 52ans±10. La (FA) était présente chez (67,4%) des hommes versus (32,6%) des femmes. La fréquence des facteurs de risque cardiovasculaires associée à (FA) était de (Figure 1): (55%) pour l'hypertension artérielle, (39%) pour le diabète, (8%) pour la dyslipidémie, (26%) pour le tabac, (30%) pour la sédentarité et (17%) pour la ménopause. 45% de nos patients étaient en insuffisance cardiaque stade III, 50 % en stade I-II de la NYHA et 5% en stade IV. L’insuffisance cardiaque était gauche dans 17,6%, droite dans 16,3% et globale dans 12%. La fréquence cardiaque moyenne était de 84,5±21 bpm. La moyenne de la pression artérielle diastolique à l’admission était de 77,4±14. La fraction d'éjection (FE) moyenne du VG à 37,5±10%. Les étiologies de l'insuffisance cardiaque chronique associées à la fibrillation atriale sont résumées dans la Figure 2.

## Discussion

La fibrillation atriale est le trouble de rythme le plus fréquent dans la pratique clinique responsable d’une morbidité significative [4]. Elle affecte 0,4-1% de la population générale et touche en particulier les sujets âgés [5]. La prévalence de la FA chez les patients en insuffisance cardiaque varie de 10 à 30%; [6] ceux qui rejoint les résultats de notre série. Plusieurs mécanismes physiopathologiques expliquent cette association entre (FA) et insuffisance cardiaque ainsi une perte de la contribution auriculaire dans le remplissage ventriculaire réduira le débit cardiaque et entrainera par conséquence une élévation de la pression artérielle moyenne précipitant l’insuffisance cardiaque chez des patients prédisposées [7]. En plus l’irrégularité et la rapidité de la fréquence cardiaque peut provoquer une dilatation du ventricule gauche avec dysfonction systolique, d’autre part l’augmentation de la pression auriculaire et de la surcharge volumique entraine un étirement auriculaire pouvant générer une FA dans l’insuffisance cardiaque [7]. La prévalence de la FA augmente proportionnellement à la sévérité de l’insuffisance cardiaque, en effet elle est < 10% chez les patients en stade I de la classification la New York Heart Association (NYHA) [8]. L’âge moyen retrouvé dans les différents registres était nettement supérieur à celui de notre série: 71.3 ans dans EHFS [9], 72.5 ans dans ADHERE et 78 ans dans OPTIMIZE-HF [10]. Ceci pourrait être expliqué par la nette prédominance de l’étiologie valvulaire rhumatismale révélée très souvent par une insuffisance cardiaque et encore fréquente dans les pays en voie de développement. En Afrique subsaharienne et dans certaines études en Asie, un jeune âge de la population ayant une FA a été noté [11, 12]. La physiopathologie et les facteurs de risque de l’insuffisance cardiaque et de la FA sont étroitement liés [13]. La FA est à la fois une cause et une conséquence d’insuffisance cardiaque. Il existe aussi différents facteurs étiologiques d’insuffisance cardiaque avec FA, dans notre série le facteur de risque cardiovasculaire le plus souvent retrouvé était l’hypertension artérielle. Elle était l’une des principales causes de (FA) selon l’étude Framingham [14]. Dans les étudesTHESUS-HF et Heart of Soweto [15], l’hypertension artérielle était un facteur étiologique prédominant d’insuffisance cardiaque. La présentation clinique d’insuffisance cardiaque dans notre série était principalement gauche, en Afrique subsaharienne c’est l’insuffisance cardiaque globale qui a été souvent relevée [16].

Nous avons réalisé l’écho doppler cardiaque chez tous nos patients, c’est un moyen de diagnostic et de suivi simple, peu couteux et reproductible. L’origine de l’insuffisance cardiaque dans notre série était surtout la pathologie valvulaire essentiellement mitroaortique le plus souvent d’origine rhumatismale. Le retard de consultation des jeunes patients et le retard de prise en charge chirurgicale étaient les deux facteurs responsables d’une évolution spontanée de la pathologie valvulaire vers l’insuffisance cardiaque et la FA. Dans les pays de l’Afrique subsaharienne, la cardiopathie hypertensive était la principale étiologie d’insuffisance cardiaque [17]. La cardiopathie ischémique constitue la deuxième étiologie en terme de fréquence dans notre série mais aussi dans les pays d’Afrique Subsaharienne, cette étiologie devient de plus en plus fréquente dans ces pays en rapport avec la notion de transition épidémiologique avec un changement de mode de vie et une urbanisation [18]. Au Maroc tout comme en Europe la pathologie ischémique est la plus fréquente cause d’insuffisance cardiaque [19], dans notre série nous avons inclus seulement les patients ayant une FA associée à l’insuffisance cardiaque ceci explique la prédominance de la pathologie valvulaire dans notre étude au dépend de la pathologie ischémique. Parmi les étiologies retrouvées, l’insuffisance cardiaque d’origine toxique retrouvée essentiellement chez les patients sous séances de chimiothérapie et/ou radiothérapie, l’amélioration des stratégies de surveillance de ce profil de patients pourrait réduire l’incidence de l’insuffisance cardiaque et améliorer le pronostic de ces patients. Nous avons aussi retrouvé un nombre non négligeable d’insuffisance cardiaque primitive, l’amélioration des moyens diagnostiques tels que l’IRM, la coronarographie et la biopsie myocardique est indispensable afin de réduire cette catégorie d’insuffisance cardiaque. La principale limite de notre étude était le recueil rétrospectif de données extraites à partir des dossiers médicaux.

## Conclusion

L’association de la fibrillation atriale et l’insuffisance cardiaque est fréquente au Maroc et en Afrique. La population concernée est jeune par rapport aux pays occidentaux. La principale cardiopathie est la valvulopathie rhumatismale. L’élaboration de stratégies visant le rhumatisme articulaire aigue est plus que nécessaire afin de réduire l’incidence de l’association (FA) et insuffisance cardiaque.

### Etat des connaissances actuelle sur le sujet

- L’épidémiologie de l’association FA et Insuffisance cardiaque en Europe et en Amérique du nord;
- Le pronostic de la FA et l’insuffisance cardiaque est défavorable;
- La cardiopathie ischémique est la plus fréquente cause.

### Contribution de notre étude à la connaissance

- Première étude au Maroc traitant l’épidémiologie de l’association entre FA Et insuffisance cardiaque;
- L’âge au Maroc est relativement jeune des patients ayant une insuffisance cardiaque avec FA;
- Nécessité d’une élaboration de stratégie prenant en charge le rhumatisme articulaire aigue.
